# Effects of Socioeconomic Status on Early Results After Sleeve Gastrectomy

**DOI:** 10.7759/cureus.81755

**Published:** 2025-04-05

**Authors:** Cagla Ozbalci, Vahit Mutlu, Mahmut Arif Yüksek, Samet Sahin

**Affiliations:** 1 Science and Technology, Bahcesehir College Science and Technology High School, Samsun, TUR; 2 General Surgery, Üsküdar University Faculty of Medicine, Istanbul, TUR; 3 General Surgery, Hitit University Faculty of Medicine, Çorum, TUR; 4 General Surgery, Ondokuz Mayıs University, Faculty of Medicine, Samsun, TUR

**Keywords:** low-income, metabolic and bariatric surgery (mbs), obesity, sleeve gastrectomy, socioeconomic status (ses)

## Abstract

Purpose

Obesity and its related metabolic diseases are a widespread public health problem worldwide. In recent years, surgical methods have played a very important role in the treatment of obesity and metabolic diseases. This study aims to investigate the effects of patients' socioeconomic status (SES) on the early results of sleeve gastrectomy (SG), which is the most preferred metabolic bariatric surgery (MBS) procedure in the world.

Methods

Data of SG patients who were operated on in the general surgery clinic of a tertiary hospital were analyzed retrospectively. A total of 322 patients who completed at least three months of follow-up after surgery were included in the study. Patients were divided into three groups - low, medium, and high - according to their SES. The effects of SES on weight loss and comorbidities associated with obesity were evaluated. The one-way analysis of variance (ANOVA), Kruksal Wallis, and chi-square tests were used in the statistical analysis of the data.

Results

The groups were homogeneous in terms of age and body mass index (BMI). There was no statistically significant difference among income groups in terms of the age, BMI, and excess weight loss (EWL) variables (p>0.05). In addition, when the relations among income groups and gender, diabetes mellitus (DM), and other diseases were examined, it was concluded that there was no relation between income status and other variables (p>0.05).

Conclusion

SES of patients has no effect on the early results of SG.

## Introduction

Metabolic bariatric surgery (MBS) procedures, which began in the mid-twentieth century with various types of intestinal bypasses, gained significant momentum after Mason EE introduced the first gastric bypass surgery. Over time, these procedures have evolved significantly [1].

Today, various MBS operations are performed worldwide, with sleeve gastrectomy (SG) representing around 60% of all obesity interventions in the United States in 2022 and approximately 70% of primary cases, excluding revision surgeries and endoscopic treatments such as balloons [2]. SG rates are similarly high globally.

Originally, SG was designed as the first step in two-stage procedures like duodenal switch and Roux-en-Y gastric bypass (RYGB) for high-risk patients with a body mass index (BMI) of >50 kg/m² [3,4]. Over time, it was understood that the procedure was very successful in both losing weight and solving the accompanying problems and was accepted as a method on its own. Fundus resection, which is a part of sleeve gastrectomy, reduces the orexigenic hormone ghrelin and thus provides sustainable weight loss [5]. Its popularity has increased considerably because it is superior to the medical treatment of type II diabetes mellitus (DM) and provides similar results to RYGB [6]. In addition to its successful results, the fact that it is also technically easier and safer compared to other MBS procedures makes it the most preferable type of surgery in the world today.

However, there are non-negligible rates of failure in all treatment methods for obesity, whether they are non-invasive methods, such as diet, exercise, medication use, and behavioral modifications; endoscopic methods, such as gastric balloons; or surgical methods. Even in malabsorptive procedures, such as RYGB, this rate reaches 40% [7,8]. Low patient motivation is shown as one of the most important reasons for failure in obesity treatment. It is important for patients to follow nutrition and exercise recommendations, especially in the postoperative period. Being able to follow ideal nutrition programs, procure recommended supplements, such as protein powder and vitamins, and participate in physical activities, such as gyms and swimming pools, are necessary for permanent success.

There are few studies on the effect of socioeconomic status (SES) on the results of sleeve gastrectomy. This study aims to explore the impact of patients' SES on the early outcomes of SG.

## Materials and methods

Study design

This study is conducted based on ethics committee approval received from OMÜ KAEK (Ondokuz Mayıs University Clinical Researches Ethics Committee) (decision number 2024/497 dated 31/12/2024). Bariatric metabolic surgery operations performed in the Department of General Surgery, Faculty of Medicine, Ondokuz Mayıs University from 2012 to the present time were retrospectively reviewed. MBS surgeries other than SG, the first 80 patients who were considered to be in the learning curve [9], and SG patients whose data could not be accessed were excluded from the study. The patients who passed at least two years after their surgery and came for a minimum three-month follow-up were included in the study. Preoperative comorbidities were DM, hypertension (HT), obstructive sleep apnea syndrome (OSAS), hyperlipidemia (HL), and thyroid function test (TFT) disorders. All surgeries were performed by the same surgeon with advanced laparoscopy experience. All surgeries were performed in reverse Trendelenburg position using 5 trocars of 5-12 mm, and the abdominal cavity was inflated with a pressure of 13-15 mmHg. Patients were divided into three groups according to their income status. In addition to EWL values, remissions of DM, HT, OSAS, HL, and TFT were also analyzed.

Definitions and measurements

Since the first two-year period after MBS was considered the early period, patients who had completed at least two years after surgery were included in the study [10]. Patients were divided into three groups according to their income status in terms of the civil servant salaries of the Republic of Turkey in the second half of 2024 [11]. Those with income below the lowest civil servant salary were classified as group 1, those with income between the lowest and highest civil servant salary were classified as group 2, and those with income above the highest civil servant salary were classified as group 3. Socio-cultural status was not taken into account when dividing into groups. Remissions were classified as complete, partial, or non-remission based on laboratory data and medication use (medicine quittance or dose reduction). The following formula was used for the calculation of excessive weight loss:

EWL percentage = (Preoperative weight - Current weight) / (Preoperative weight - Ideal weight) x 100

The Miller formula was used to calculate the ideal body weight [12]. Accordingly, fixed MBI was not preferred to be evaluated and ideal weight was calculated individually for each patient.

Statistical analysis

The IBM SPSS v 23 program (IBM Corp., Armonk, NY, US) and Microsoft Excel 2010 program (Microsoft Corporation, Redmond, WA, US) were used for the statistical analysis of the data obtained from the research. The Kolmogorov-Smirnov test was used to test the conformity of the data to normal distribution. The one-way analysis of variance (ANOVA) and Kruskal-Wallis tests were used to compare the means of independent groups with more than two categories, and the chi-square independence test was used to test the independence of categorical variables.

Categorical data were compared using the chi-square test using cross-tables. Since the data numbers were low in comorbidities other than DM, they were combined under the other diseases variable (in the chi-square test, if 20% of the expected frequencies in the rxc order tables are less than 5, cell merging is used). The one-way ANOVA and Kruksal-Wallis tests were performed to determine whether there were differences in age, BMI, and EWL values ​​between the groups of the income variable.

## Results

A total of 322 patients (217 (67.4%) female and 105 (32.6%) male) were included in the study. The mean age was 36 ± 10.85 years, and the mean BMI was 46± 7.10 kg/m². A total of 149 (46.2%) patients had the following comorbidities: DM (n:81, 25.2%), HT (n:57, 17.7%), OSAS (n:12, 3.7%), HL (n:53, 16.5%), or abnormal TFT (n:34, 10.5%). 304 (94.4%) with 6-month EWL, 299 (92,8%) with 12-month EWL, and 293 (90.9%) with 24-month EWL were included in the study. Irrelevant parameters, such as complications, duration of surgery, and hospital stay, were not evaluated.

There was no statistically significant difference among the groups according to age and BMI changes (p=0.681 and p=0.977, respectively). There was no significant difference among the median %EWL values ​​of the groups at three months, six months, one year, and two years (p>0.05). Although EWL values ​​increased according to the months, this increase did not cause any difference among the groups (Table 1).

**Table 1:** Comparison of groups according to quantitative variables x̄±σ: mean± standard deviation, *Kruskal Wallis, **one-way ANOVA (analysis of variance) (Group 1: Income below the lowest civil servant salary, Group 2: Income between the lowest and highest civil servant salary, Group 3: Income above the highest civil servant salary)

	Group 1			Group 2			Group 3			p
	n	x̄±σ	Median	n	x̄±σ	Median	n	x̄±σ	Median	
			(25*percentile-75*percentile)			(25*percentile-75*percentile)			(25*percentile-75*percentile)	
Age	99	37.07±12.08	35	142	35.4±10.46	34	81	35.9±9.98	35	0.681*
			(27-47)			(28-42)			(29-41)	
BMI	99	46.43±8.21	45.3	142	45.8±6.76	44.05	81	45.72±6.28	44.4	0.977*
			(40.4-50.7)			(41.1-49.07)			(40.85-49.2)	
3-month %EWL	99	45.18±11.21	42.8	142	44.43±9.29	43.8	81	44.15±9.41	44	0.764**
			(37.7-50)			(38.35-49.47)			(37.5-49.15)	
6-month %EWL	95	66.31±15.56	62.6	134	65.16±14.1	64.8	75	65.48±13.88	63.9	0.839**
			(55.2-74.7)			(56.3-74.52)			(56.6-74.2)	
1-year %EWL	90	84.31±18.19	81.9	134	82.55±17.08	83	75	82.61±16.75	82.7	0.731**
			(71.97-96.35)			(71.27-95.02)			(72.2-93)	
2-year %EWL	88	89.97±18.89	86.6	131	84.97±78.39	86.9	74	87.01±17.11	86.9	0.141**
			(77.42-104.77)			(76.2-98.6)			(77.47-99.87)	

**Figure 1:**
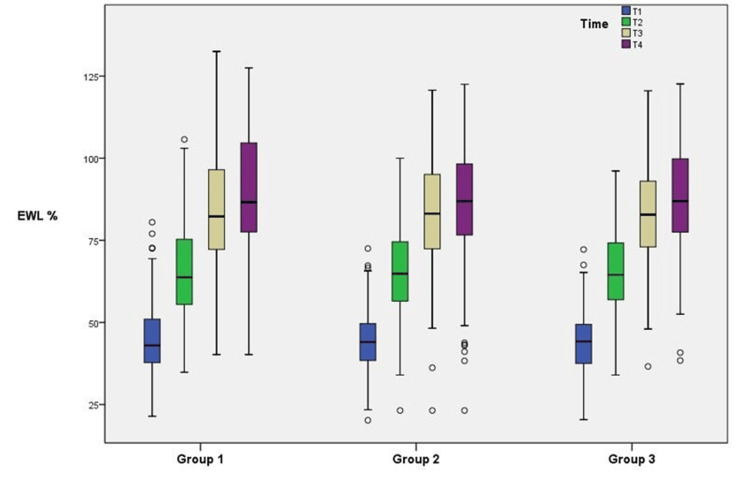
Box plot of % EWL values according to group and time EWL: excess weight loss

The one-way ANOVA and Kruskal-Wallis tests were performed to determine whether there were differences among the groups of the income status variable according to age, BMI, and EWL values. In Figure 1, T1, T2, T3, and T4 show the EWL values ​​of the third month, sixth month, first year, and second year, respectively.

The fact that the EWL values ​​of the income groups are close to each other in the box plot graphical representation indicates a similar distribution.

There was no significant difference in DM remission among the three groups (p>0.05). Categorical data were compared using the chi-square test using cross-tables. Since the data numbers were low in comorbidities other than DM, they were combined under the other diseases variable (in the chi-square test, if 20% of the expected frequencies in the rxc order tables are less than 5, cell merging is applied).

When the table is examined, it is concluded that there is no relation between income groups and gender, DM and other diseases, and income status and other variables (p>0.05) (Table 2).

**Table 2:** Comparison of groups according to qualitative variables *Chi-square test, other remissions: HT, OSAS, HL, TFT There was no statistically significant difference between the groups (p>0.05) HT: hypertension; OSAS: obstructive sleep apnea syndrome; HL: hyperlipidemia; TFT: thyroid function tests

Variable	Group 1: n (%)	Group 2: n (%)	Group 3: n (%)	Total: n (%)	df	Test Statistics*	p*
Gender							
Female	72 (72.7%)	95 (66.9%)	50 (61.7%)	217 (67.4%)	2	2.48	0.289
Male	27 (27.3%)	47 (33.1%)	31 (38.3%)	105 (32.6)			
DM Remission							
Partial	21 (80.8%)	33 (94.3%)	19 (95%)	73 (90.1)	2	3.771	0.152
Complete	5 (19.2%)	2 (5.7%)	1 (5%)	8 (9.9%)			
Other Remission							
Partial	17 (27.9%)	19 (31.1%)	9 (26.5%)	45 (28.8%)	4	0.786	0.94
Complete	39 (63.9%)	39 (63.9%)	23 (67.6%)	101 (64.7%)			
No Remission	5 (8.2%)	3 (4.9%)	2 (5.9%)	10 (6.4%)			

## Discussion

Obesity is one of the most important health problems of our time. Since 1980, the number of people classified as overweight and obese has doubled, and today, one-third of people are in the overweight and obese category [13]. Obesity should not be seen as just a cosmetic problem. It is the leading cause of preventable death, along with smoking [14]. It has long been known that it is associated with type II DM, HT, HL, OSAS, cardiovascular diseases, nonalcoholic fatty liver, arthritis, choledocholithiasis, and many types of cancer [15].

People with a BMI>40kg/m² who have previously tried nonsurgical methods, such as diet and exercise, who are not addicted to drugs or alcohol, who do not have endocrinological diseases, and who are psychologically stable are candidates for bariatric surgery. In addition, those with a BMI of 35-40 kg/m² who are expected to improve with weight loss and who have insulin resistance, DM, fatty liver, HT, some cardiovascular diseases, and various joint problems are candidates for MBS [16]. In recent years, MBS has been recommended for type II DM patients with a BMI>30 kg/m² who have not benefited despite diet, medical treatment, and lifestyle changes [17].

It is an indisputable fact that MBS is the most effective method in the treatment of obesity today. The success of surgeries has not been achieved with any pharmacological treatment [18]. However, it is known that even surgical treatment is not a definitive solution for some patients. Because there are some facts that even surgery cannot change. First of all, obesity is a chronic disease. The World Health Organization (WHO) defines chronic diseases as long-term, slowly progressing, and non-contagious diseases [19]. As can be understood from this definition, chronic diseases are diseases that generally do not have a definitive cure and progress with relapses. In other words, obesity is a disease that tends to relapse when left alone by its nature. In addition, genetic factors play a role in the development of obesity at a high rate of 80% [20]. This means that no treatment method can eliminate the cause of the disease in most patients. All of these are indicators that even surgery alone will not be sufficient for treatment and that patient compliance and effective cooperation between patient and physician are necessary for a successful result.

After bariatric metabolic surgery, it is necessary to pay attention to some issues such as getting quality sleep, consuming enough water, eating protein-based foods, taking multivitamin supplements, doing enough exercise, chewing food well, and separating solids and liquids in meals. For success, it is important for patients to make a "lifestyle change" in this direction in the post-surgery period, and especially to comply with the process in terms of nutrition and exercise. It is recommended to take the necessary care in issues such as being able to follow ideal nutrition programs, taking recommended supplements, such as protein powder and multivitamins, and being able to participate in physical activities such as the gym and swimming pool for permanent success.

Many factors, such as the time elapsed after surgery, age, gender, education level, occupation, and especially compliance postoperatively are important for success after MBS [21]. The effects of different education levels, occupations, and income status on patients' quality of life are known. Low education levels and low income levels negatively affect compliance with the postoperative process. In fact, it is reported that the results obtained in studies conducted on this subject can be used to customize follow-ups by paying special attention to patients with lower SES [22].

Proper nutrition after MBS is extremely important for healthy weight loss. It is not possible to get enough calories and protein from food, especially in the first months after surgery, since mostly liquids are consumed. Protein is one of the most important macronutrients needed after MBS, and inadequate intake can affect the process of expected weight loss and lean mass loss. Studies have shown that protein intake after MBS not only increases satiety but can also change long-term surgical results in terms of weight and fat loss. It is suggested that individuals who consume more dietary protein and less fat lose more weight for 10 years after MBS. Having access to protein supplements is necessary to lose weight by preserving muscle mass and losing fat during this period of rapid weight loss. In other words, if protein powders are not used as support, it will be difficult to maintain the anatomical, physiological, and hormonal integrity of the body [23]. In addition, mineral and multivitamin supplements are also recommended to prevent micronutrient deficiency, especially during the first year after SG. Studies show that reasons for not using supplements include forgetting to use them, not using them because of side effects or not liking the taste, as well as not being able to access these products due to economic reasons [24].

Post-MBS exercise programs are believed to be an integral part of the treatment for weight management, increasing weight loss, maintaining ideal body weight, and preventing weight regain, as well as reducing cardiovascular and metabolic risk factors and improving quality of life [25]. Since physical activity has positive cardiovascular and metabolic benefits independent of surgery and weight loss, it is considered essential for the treatment of morbid obesity and optimization of MBS results [23,25]. According to a study analyzing the physical activity processes of MBS patients, it was emphasized that economic factors are also important in the sustainability of these processes [26].

Studies show that exercising patients lose more weight than non-exercising ones. Exercise is associated with more weight loss, particularly 12-24 months after MBS. While studies support the inclusion of physical activity as part of the postoperative care of MBS patients, the intensity, duration, and optimal type of exercise have not yet been determined. Current exercise recommendations derived from a comprehensive review of data from non-surgical patients by the American College of Sports Medicine (ACSM), recommend at least 30 minutes of exercise per day and 150 minutes per week. However, the ACSM recommends at least 60 minutes of moderate-intensity exercise, 5 days per week, or 300 minutes per week to achieve significant weight loss. MBS patients who exercised at least 150 minutes per week also lost significantly more weight than those who did not. Those who decreased their physical activities were 2.3 times more likely to fail. It is quite possible that patients who choose to exercise more are more motivated to adhere to a postoperative diet program than those who do not exercise [27,28].

Regular follow-up appointments after bariatric metabolic surgery are critical for evaluating the effectiveness of the operation, ensuring proper nutrition, increasing patient motivation, and quickly identifying any potential complications. It is known that patients who comply with follow-ups are more successful. Studies aiming to determine compliance with follow-up appointments after MBS and to examine factors associated with compliance show that demographic and socioeconomic factors have an undeniable importance in compliance with follow-ups. Race, insurance type, distance to the hospital, education, and income status are determinative in this regard [29,30].

In the USA alone, 315 billion dollars is spent annually on medical costs of obesity in adult patients [23]. It is known that SES is associated with obesity. Systematic reviews and meta-analyses show that subgroups with easy access to cheap and unhealthy foods and inadequate physical activity are at risk starting from their childhood [31]. MBS is the most effective method for treating obesity and can play an important role in reducing the direct and indirect costs of obesity treatment. However, patients need to comply with the post-operative process to benefit maximally from MBS. Studies show that patients who change their lifestyle, apply a proper diet, use protein and multivitamin supplements, do regular exercise, and comply with follow-up programs are more successful in the long term. It is known that occupation and income status are important in successfully managing the process and that low-income occupation groups are associated with failure [22]. In other words, SES is associated with obesity as well as long-term success after MBS.

A study examining the effects of making a lifestyle change in the first 12 months after MBS on weight loss or health outcomes showed that this intervention did not make a significant difference [32]. However, in addition to SG, surgeries with different mechanisms of action, such as RNYGB and mini gastric bypass, were also included in this study. We believe that including only SG in our study is more meaningful in terms of ensuring standardization. At the same time, since the first two years after MBS are scientifically accepted as the early period, we believe that examining the first two-year results is more meaningful to evaluate the early period [10].

The first two years after MBS are reported as the early period, two to five years as the middle period, and after five years as the late period. Our study has a two-year follow-up period and was evaluated only according to the early-period follow-ups. Evaluating the middle and late period results may lead to more accurate results. For this purpose, we are currently conducting a study that we plan to present to you in the near future. In addition, since our study is retrospective, patients whose data we could not access and who did not come to their follow-ups were excluded from the study. However, the fact that this patient group did not have their follow-ups may be related to their socioeconomic status. Therefore, a prospective multicenter study that includes all patients who underwent SG and has more patients may provide more accurate results.

## Conclusions

The first period after bariatric metabolic surgery is the period when both the restrictive and hormonal effects of the surgeries are felt the most. In other words, a certain level of weight loss and metabolic improvement is expected, regardless of the factors that the patients have. According to the literature, SES also has a role in factors related to success such as nutrition, exercise, and compliance with follow-up programs. In our study, we aimed to determine whether SES makes a difference in the early period when a certain level of improvement is expected. For this purpose, we divided the patients who had undergone SG into three groups according to their SES. We statistically evaluated both the weight loss and the remission of obesity-related comorbidities. We concluded that SES was not effective in success after SG in the first two years. However, it is known that these factors are important in long-term success after MBS. Therefore, studies investigating the long-term effects of SES on SG are needed.
